# High-Throughput Sequencing Decodes tsRNA Landscapes: Insights into Cancer Biomarkers and Therapeutic Targets

**DOI:** 10.3390/ijms27041949

**Published:** 2026-02-18

**Authors:** Miaoyan Pu, Luyu Shi, Chuanlin Shen, Haimei Cheng, Weijie Ding, Jiaxin Tian, Junhong Ye, Youquan Bu, Ying Zhang

**Affiliations:** 1 Department of Biochemistry and Molecular Biology, School of Basic Medicine, Chongqing Medical University, Chongqing 400016, China; 2023110030@stu.cqmu.edu.cn (M.P.); 2023110032@stu.cqmu.edu.cn (L.S.); 2024110036@stu.cqmu.edu.cn (C.S.); 2024110024@stu.cqmu.edu.cn (H.C.); 2025110031@stu.cqmu.edu.cn (W.D.); yejunhong@cqmu.edu.cn (J.Y.); 2 Molecular Medicine and Cancer Research Center, Chongqing Medical University, Chongqing 400016, China; 3 The First Clinical College, Chongqing Medical University, Chongqing 400016, China; 2021220654@stu.cqmu.edu.cn

**Keywords:** tsRNAs, high-throughput sequencing, cancer, biomarker, therapeutic target

## Abstract

Transfer RNA-derived small RNAs (tsRNAs) represent an emerging category of small non-coding RNAs generated through specific cleavage of precursor or mature tRNAs. Increasingly recognized as pivotal players in the pathogenesis of complex malignancies, tsRNAs not only regulate cancer progression but also hold promising clinical potential for cancer diagnosis and treatment. This review highlights recent advances in the application of high-throughput sequencing technologies in the systematic identification of tsRNAs, with a focus on their roles in cancer diagnosis, prognostic assessment, and targeted therapy. Delving into the translational medicine dimensions of tsRNAs may provide novel strategies for molecular diagnosis and therapeutic interventions in oncology.

## 1. Introduction

**Figure 1:**
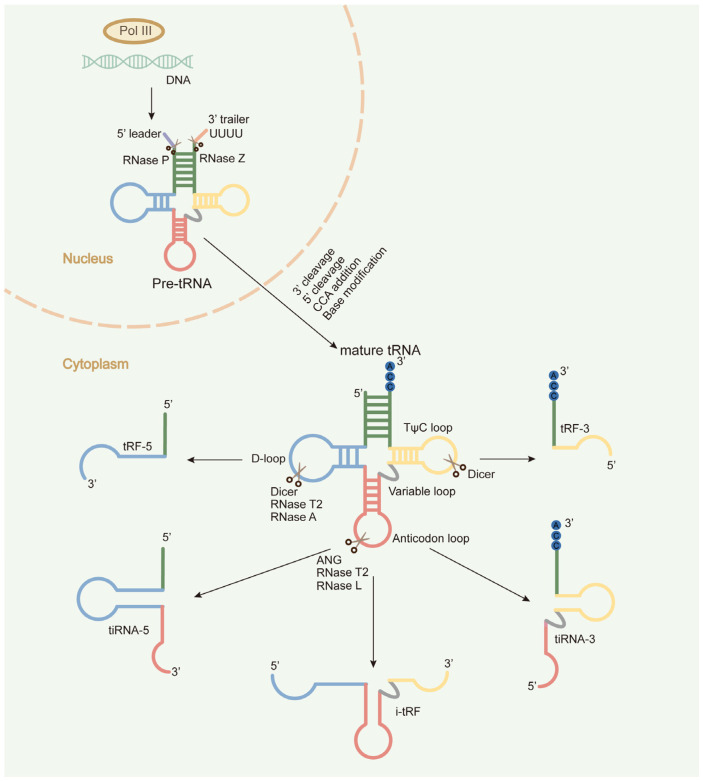
Biogenesis and classification of tsRNAs. In the nucleus, RNA polymerase III catalyzes the transcription of tRNA genes to produce precursor tRNAs, which undergo post-transcriptional processing to form mature tRNAs. Specific ribonucleases cleave mature tRNAs at distinct sites, generating various types of tsRNAs, including tRFs, tiRNAs, and i-tRFs.

Transfer RNAs (tRNAs) are a class of non-coding RNAs that can specifically recognize codons on mRNA and deliver the corresponding amino acids to ribosomes during translation. This canonical function of tRNA is intrinsically dependent upon its highly conserved structure. In eukaryotic cells, precursors tRNAs (pre-tRNAs) are transcribed by the action of RNA polymerase III and then processed through post-transcriptional modifications to yield mature tRNAs. Mature tRNAs exhibit a cloverleaf-shaped secondary structure comprising four characteristic loops: the D loop, the TψC loop, the anticodon loop, and the variable loop. This secondary structure further folds into an inverted L-shaped tertiary architecture stabilized by an intricate network of hydrogen bonds [1]. Beyond its canonical role as a molecular adaptor in translation, tRNA has diverse non-canonical functions, including the biogenesis of small RNAs with regulatory functions. Specifically, precursor or mature tRNAs can be cleaved by dedicated ribonucleases under particular conditions to generate a family of functional small non-coding RNAs (sncRNAs) [2,3,4,5], known as tRNA-derived small RNAs (tsRNAs) [5,6,7,8,9,10,11]. Based on distinct cleavage sites within mature tRNAs, tsRNAs are categorized into 5′-tsRNAs, 3′-tsRNAs, and internal tsRNAs [8,12,13]. As depicted in Figure 1, 5′-tsRNAs originate from the 5′ terminus of mature tRNAs and can be further subdivided into tRF-5, which are produced through the cleavage of the D loop by endonucleases such as Dicer, RNase T2, or RNase A; and tiRNA-5s, which are produced under stress conditions through the cleavage of the anticodon loop by endonucleases including angiogenin (ANG), RNase T2, and RNase L. Similarly, 3′-tsRNAs are derived from the 3′ terminus of mature tRNAs and classified as tRF-3s (cleaved near the TψC loop) or tiRNA-3s (cleaved at the anticodon loop).

**Figure 2:**
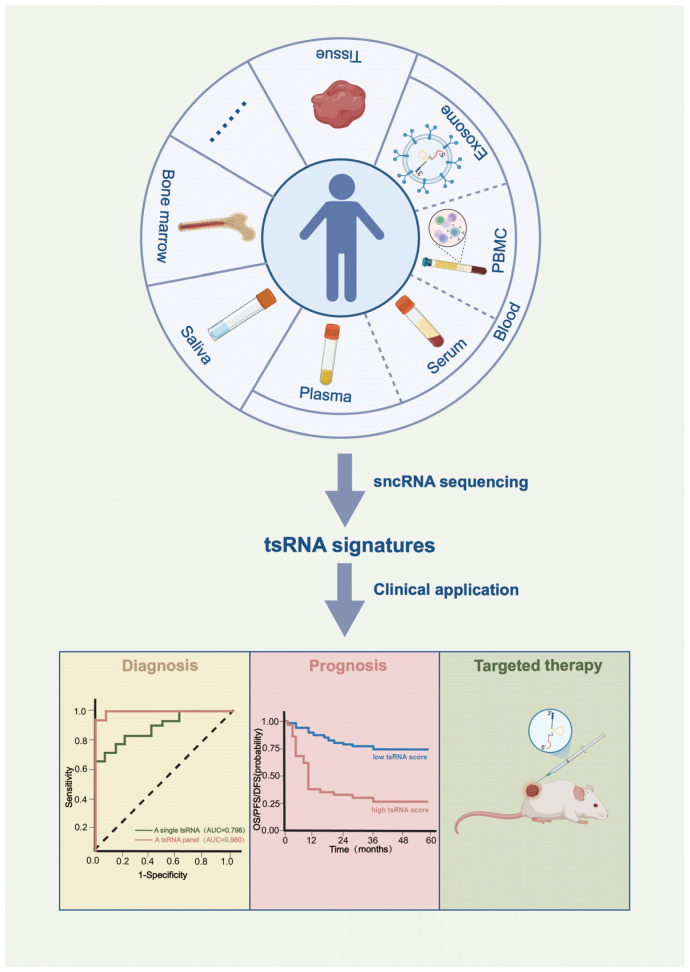
Sample types for tsRNA sequencing and clinical applications in cancer. tsRNAs are widely present in a variety of human tissues and biofluids, including plasma, serum, peripheral blood mononuclear cells (PBMCs), exosomes, saliva, and bone marrow. They exhibit aberrant expression in cancer and contribute to the cancer progression, demonstrating considerable potential as diagnostic biomarkers, prognostic indicators, and therapeutic targets in oncology.

Through their sequence characteristics, modification patterns, and structural features, tsRNAs exert context-dependent functions as either tumor suppressors or promoters during tumorigenesis through multiple mechanisms, involving miRNA-like regulatory pathways, the mode of RNA G-quadruplex (RG4), tsRNA modification-specific interactions, alteration of mRNA secondary structure, and ribosome binding [12]. The functional role of tsRNAs in tumorigenesis was first documented in prostate cancer research [4]. Anindya Dutta’s group utilized high-throughput RNA sequencing to identify the tsRNA expression profiles of prostate cancer cell lines and found that tRF-1001 (derived from ELAC2-mediated cleavage of pre-tRNAser) facilitated G2/M transition to promote prostate cancer cell proliferation. Several years later, Carlo M. Croce and Yuri Pekarsky’s group conducted a series of systemic studies demonstrating the significant involvement of tsRNAs in tumorigenesis [14,15,16]. Their investigation in B-cell chronic lymphocytic leukemia (CLL) revealed that tsRNA-53 (initially named miR-3676) suppressed the expression of the oncogene TCL1 by targeting its 3′ untranslated region (UTR). Reduced tsRNA-53 expression led to elevated TCL1 expression, thereby driving CLL progression [14]. In subsequent studies, they obtained the tsRNA expression profiles of CLL, lung cancer, breast cancer, ovarian cancer, and colorectal cancer using microarray analysis. Through this approach, they identified differentially expressed tsRNAs. For instance, tsRNA-46, tsRNA-47, and tsRNA-53 exhibited downregulation in both CLL and lung cancer, and all of them could repress the colony formation capacity of lung cancer cells [15,16]. As yet, accumulating studies have uncovered the aberrant expression and regulatory roles of tsRNAs in cancer [17,18,19,20,21,22,23,24,25,26,27,28,29,30], providing compelling evidence for their potential as diagnostic biomarkers, prognostic indicators, and therapeutic targets. Next-generation deep sequencing is the premier choice for high-throughput tsRNA profiling. As summarized in Figure 2, the following sections will highlight recent advances leveraging sequencing technologies to identify clinically relevant tsRNAs for cancer diagnosis, prognosis, and targeted therapy.

## 2. tsRNAs as Potential Diagnostic Biomarkers for Cancer

The primary sample types for tsRNA sequencing encompass clinical tissue samples and body fluid samples.

### 2.1. Tissue Samples

The repertoire of functional tsRNAs in tumor tissues enables tissue-derived tsRNA profiling to effectively identify cancer-associated aberrant tsRNA signatures, representing promising diagnostic biomarker candidates. Previous studies have employed traditional RNA sequencing to delineate tissue-specific tsRNA expression landscapes across multiple malignancies, including lung cancer, colorectal cancer (CRC), gastric cancer, and kidney cancer [22,31,32,33,34] (Table 1). Wang et al. performed conventional RNA sequencing on paired tumor tissues and adjacent normal tissues from five early-stage lung adenocarcinoma patients, identifying nine differentially expressed tsRNAs. Subsequent quantitative reverse-transcription PCR (qRT-PCR) validation in a discovery cohort revealed the serum levels of five tsRNAs (including tRF-Ser-TGA-003, tRF-Val-CAC-005, tRF-Ala-AGC-060, tRF-Val-CAC-024, and tiRNA-Gln-TTG-001) were significantly elevated in patients with malignant pulmonary nodules relative to that of patients with benign pulmonary nodules. A combined diagnostic model incorporating the serum levels of these five tsRNAs and CT imaging features was developed. Further evaluation across discovery, internal validation, and external validation cohorts demonstrated robust performance in distinguishing benign from malignant pulmonary nodules, achieving an area under the curve (AUC) of 0.837 with 85.7% sensitivity and 84.0% specificity [31]. Lu et al. similarly leveraged conventional RNA sequencing to characterize tsRNA expression in paired tumor tissues and adjacent normal tissues from 10 CRC patients, as well as in plasma exosomes from both these patients and 10 healthy individuals. qRT-PCR validation confirmed significant upregulation of tRF-3022b, tRF-3030b, and tRF-5008b in both tumor tissues and patient-derived plasma exosomes. These exosomal tsRNAs achieved moderate diagnostic accuracy with AUCs of 0.768 (tRF-3022b), 0.748 (tRF-3030b), and 0.692 (tRF-5008b) in discriminating CRC patients from healthy individuals [32]. Collectively, these findings demonstrate that differentially expressed tsRNAs identified in tumor tissue samples can also exhibit their dysregulation in circulating serum/plasma samples, suggesting their potential as non-invasive diagnostic biomarkers.

Notably, traditional RNA sequencing cannot accurately capture tsRNA expression profiles, as the standard library preparation protocol for small RNA sequencing is incompatible with tsRNAs. Firstly, tsRNAs possess unique terminal structures such as 5′-OH, 3′-P, and 2′,3′-cP [28,35,36], which hinder the ligation of 5′ and 3′ adapters during library preparation. Secondly, as tsRNAs originate from tRNAs, they also carry numerous modifications (e.g., m^1^A, m^3^C, m^1^G, and m_2_^2^G), which impede the reverse transcription in library preparation [35,36,37,38]. Consequently, the standard small RNA library construction workflow fails to generate cDNA templates from tsRNAs due to their unique structural features, resulting in the systematic omission of these specially modified tsRNAs in sequencing datasets. Recognizing the inherent bias of conventional RNA sequencing against modified sncRNAs, investigators are now implementing optimized RNA sequencing workflows, which involve the removal of RNA modifications prior to library construction, to achieve more comprehensive tsRNA landscape in tumor tissue samples [23,39,40,41,42,43,44,45,46] (Table 1). For instance, Mo and colleagues first utilized a commercial kit to eliminate both terminal special modifications and internal methylation modifications from RNAs, followed by cDNA library preparation and RNA sequencing. This approach enabled the acquisition of tsRNA expression profiles from paired tumor tissues and adjacent normal tissues in six patients with breast cancer, ultimately identifying 30 differentially expressed tsRNAs. Subsequent validation using qRT-PCR confirmed the downregulated expression of 5′-tiRNA^Val^ in tumor tissues. Parallel serum analysis revealed markedly reduced levels of 5′-tiRNA^Val^ in patients with breast cancer compared with healthy controls (AUC = 0.756, 90.0% sensitivity and 62.7% specificity for disease discrimination) [23]. Our research group recently adopted PANDORA-seq to comprehensively profile sncRNAs by eliminating methylation and terminal modifications. Through systematic analysis of sncRNA expression signatures in tumor tissues and adjacent normal tissues from five hypopharyngeal carcinoma patients, we identified 4798 significantly differentially expressed sncRNAs, including miRNAs, tsRNAs, and rsRNAs. Subsequent validation revealed eight sncRNAs as potential diagnostic biomarkers for hypopharyngeal carcinoma [46].

### 2.2. Peripheral Blood Samples

Liquid biopsy, a minimally invasive diagnostic approach, enables the comprehensive profiling of tumor-derived biomarkers, including circulating tumor cells, cell-free nucleic acids, and exosomes, in biofluids such as blood, urine, or cerebrospinal fluid. Through serial sampling, it facilitates real-time monitoring of tumor progression, longitudinal assessment of therapeutic response, and early detection of minimal residual tumor foci, thereby supporting precision oncology [47,48,49]. tsRNAs exhibit remarkable stability in biofluids due to extensive modifications [50]. They evade degradation by ribonucleases in biofluids through multiple protective mechanisms, including encapsulation within extracellular vesicles (EVs) [51], binding to ribonucleoproteins [52], and formation of specialized higher-order structures [11,53,54]. Beyond serving as disease biomarkers, tsRNAs in biofluids exert active biological functions [8,51]. Emerging evidence has demonstrated that paternal dietary interventions altered the tsRNA landscape within epididymal epithelial cells, triggering the selective packaging of diet-responsive tsRNAs into epididymosomes for intercellular transfer to mature sperm cells. These paternally inherited tsRNAs mediated transgenerational epigenetic inheritance during fertilization, where they orchestrated embryonic gene expression reprogramming [55,56,57,58]. Moreover, T cells secreted immunosuppressive tsRNAs via EVs to potentiate T cell activation [59], and these tsRNA-enriched EVs might mediate intercellular communication to modulate recipient cell functions.

Peripheral blood represents the most widely utilized biospecimen for liquid biopsy, and cell-free nucleic acids in peripheral blood are the optimal choice for tsRNA biomarker discovery owing to their relatively simple extraction protocols. Through an optimized RNA sequencing pipeline, researchers have successfully profiled the expression signatures of circulating tsRNAs in peripheral blood from patients with pancreatic cancer, lung cancer, colorectal cancer, breast cancer, and ovarian cancer [60,61,62,63,64,65] (Table 1). For instance, Yang et al. examined the tsRNA expression profiles of paired pre- and post-operative plasma samples from 9 patients with non-small cell lung cancer (NSCLC), identifying 8 differentially expressed tsRNAs. Subsequent validation using qRT-PCR demonstrated that AS-tDR-007333 was significantly upregulated in pre-operative samples compared to both post-operative samples and healthy controls. AS-tDR-007333 achieved robust diagnostic potential with an AUC of 0.9379 for distinguishing NSCLC patients from healthy individuals at 97.78% sensitivity and 79.31% specificity [61]. Critically, diagnostic panels incorporating multiple tsRNA biomarkers and others demonstrate superior diagnostic performance over individual tsRNA markers. Jin and colleagues systematically profiled tsRNA expression patterns in serum samples from patients with pancreatic cancer and healthy controls, identifying 26 differentially expressed tsRNAs. Using TaqMan-based qRT-PCR assays, they confirmed significant upregulation of tRF-Pro-AGG-004 and tRF-Leu-CAG-002 in the serum of patients with pancreatic cancer compared to healthy individuals. In a large cohort of 204 patients with pancreatic cancer and 154 healthy controls, the dual-tsRNA signature outperformed individual tsRNA markers and conventional serum protein biomarkers (CA19-9 and CEA), achieving exceptional diagnostic accuracy (AUC = 0.94) with 85% sensitivity at 96.4% specificity [60]. Wang and colleagues analyzed the tsRNA expression profiles of plasma samples from 8 patients with breast cancer and 4 healthy controls, followed by validation using qRT-PCR. It revealed that the levels of tRF-Arg-CCT-017, tRF-Gly-CCC-001, and tiRNA-Phe-GAA-002 were remarkably higher in both plasma and plasma-derived exosomes from patients with cancer compared to healthy individuals. The combined model incorporating these three tsRNAs demonstrated superior diagnostic performance over individual tsRNA markers [64]. Guo’s research team leveraged SLiPiR-seq (an RNA sequencing method addressing RNA terminal modification challenges) to profile plasma RNA signatures across 139 patients with lung cancer and 106 healthy controls. They developed a multi-classifier model (mRNA/snRNA/snoRNA/tsRNA) that achieved 99.28% sensitivity and 100% specificity in the discovery cohort, and 76.92% sensitivity and 95.24% specificity in the validation cohort [62].

In addition to their free form in peripheral blood, tsRNAs are also encapsulated within blood-borne exosomes. Exosomes, approximately 100 nm extracellular vesicles, are generated from the inward budding of endosomal membranes to form multivesicular bodies. The fusion of multivesicular bodies with the cell membrane results in exosome secretion. Exosomes carry a variety of bioactive molecules, including proteins, lipids, DNA, and RNA, with tsRNAs constituting an important functional component of this vesicular RNA repertoire. Noteworthy, the molecular heterogeneity of exosomes is intrinsically linked to their cellular origins and disease-specific contexts, underscoring exosomal tsRNAs as promising candidates for tumor diagnosis and targeted therapy [66,67]. For instance, Zhu et al. examined the tsRNA expression profiles in plasma exosomes from five hepatocellular carcinoma (HCC) patients and five healthy controls using conventional RNA sequencing. They identified 46 differentially expressed tsRNAs, among which tRNA-ValTAC-3, tRNA-GlyTCC-5, tRNA-ValAAC-5, and tRNA-GluCTC-5 were further verified to be significantly upregulated in HCC-derived plasma exosomes using qRT-PCR [68]. As outlined above, Lu et al. conducted a comprehensive tsRNA expression profiling of plasma exosomes from 10 CRC patients and 10 healthy controls using conventional RNA sequencing. Further validation through qRT-PCR confirmed the significant upregulation of tRF-3022b, tRF-3030b, and tRF-5008b in CRC-derived plasma exosomes. The AUC values for distinguishing CRC patients from healthy controls were as follows: 0.7684 (tRF-3022b), 0.7485 (tRF-3030b), and 0.6921 (tRF-5008b) [32].

**PBMCs** participate in systemic immune responses during tumor progression, and characteristic alterations in PBMCs can reflect tumor progression and immune status in patients, thereby aiding in cancer diagnosis and therapy [69,70,71,72]. Utilizing traditional RNA sequencing to detect the sncRNA expression profiles of PBMCs from 36 patients with lung cancer, 10 pulmonary tuberculosis patients, and 13 healthy controls, Gu and colleagues identified a novel TRY-RNA signature comprising 9 tsRNAs, 8 rsRNAs, and 8 ysRNAs. The TRY-RNA signature demonstrated superior diagnostic performance compared to miRNA-based signatures, achieving excellent discrimination in the discovery cohort (AUC = 1) and the validation cohort (AUC = 0.93) for distinguishing patients with lung cancer from non-cancer individuals [73].

### 2.3. Others

Saliva, cerebrospinal fluid, ascites, pleural effusion, urine, etc., also offer biological reservoirs for tsRNA biomarker discovery [47]. For example, Li and colleagues conducted in-depth sncRNA expression profiling of salivary exosomes from three esophageal squamous cell carcinoma (ESCC) patients and three healthy subjects using traditional RNA sequencing. They identified 32 significant upregulated small RNAs in ESCC-derived salivary exosomes. Of these, tRNA-GlyGCC-5 and sRESE (a previously uncharacterized small RNA) were verified to be elevated in the ESCC cohort. The AUC values were 0.878 for tRNA-GlyGCC-5 and 0.871 for sRESE for discriminating tumor patients from healthy subjects. Importantly, a combinatorial model incorporating both small RNAs exhibited superior diagnostic performance (AUC = 0.933), yielding 90.50% sensitivity and 94.20% specificity [74]. Xia et al. adopted traditional RNA sequencing to analyze the sncRNA expression profiles in both serum and bone marrow samples from 80 acute myeloid leukemia (AML) patients and 12 healthy controls. Their analysis revealed that the tsRNA signature exhibited superior diagnostic performance compared to the miRNA signature in distinguishing AML patients from healthy individuals [75].

**Table 1:** Sequencing-based identification of tsRNAs with clinical utility in cancer.

Cancer Type	Sequencing Sample Type	Sequencing Method	Potential Clinical Utility			References
			Diagnosis	Prognosis	Targeted Therapy	
HCC	Tissue	Optimized-seq	√		√	[43]
	Tissue	Traditional-seq	√			[76,77]
	Tissue	Optimized-seq	√			[78]
	Serum	Traditional-seq	√	√		[79]
	Plasma exosome	Traditional-seq	√			[68]
Lung cancer	Tissue	Traditional-seq	√			[31]
	Plasma	Optimized-seq	√		√	[61]
	Plasma	SLiPiR-seq	√			[62]
	Serum	Optimized-seq	√			[80]
	PBMC	Traditional-seq	√			[73]
CRC	Tissue	Optimized-seq	√		√	[39,81]
	Tissue	Optimized-seq	√	√	√	[82]
	Tissue Plasma exosome	Traditional-seq	√		√	[32]
	Plasma	Optimized-seq	√			[63]
Breast cancer	Tissue	Optimized-seq	√			[23,44]
	Plasma	Optimized-seq	√	√		[64]
	Cell line	Traditional-seq			√	[20,29]
Gastric cancer	Tissue	Traditional-seq	√		√	[22]
	Tissue	Optimized-seq	√		√	[42]
Thyroid cancer	Tissue	Optimized-seq	√		√	[40,45]
Pancreatic	Tissue	Optimized-seq	√	√	√	[17]
	Serum	Optimized-seq	√	√	√	[60]
RCC	Tissues	Traditional-seq	√			[33]
	Tissue Plasma	Optimized-seq	√			[83]
ESCC	Saliva exosome	Traditional-seq	√	√		[74]
Ovarian cancer	Serum	Traditional-seq	√			[65]
Prostate cancer	Tissue	Traditional-seq	√	√		[84]
LSCC	Tissue	Optimized-seq	√		√	[41]
Bladder cancer	Plasma	Traditional-seq	√			[85]
AML	Serum Bone marrow	Traditional-seq	√	√		[75]
MM	Serum exosome	Optimized-seq	√			[86]

HCC: Hepatocellular carcinoma; CRC: Colorectal cancer; RCC: Renal cell carcinoma; ESCC: Esophageal squamous cell carcinoma; LSCC: Laryngeal squamous cell carcinoma; AML: Acute myeloid leukemia; MM: Multiple myeloma.

## 3. tsRNAs as Potential Prognostic Biomarkers for Cancer

High-throughput sequencing technologies have been widely utilized to identify tumor-associated tsRNAs with diagnostic value, while parallel investigations have systematically assessed their prognostic potential across multiple malignancies, including pancreatic cancer, ESCC, lung cancer, breast cancer, CRC, and AML [17,60,61,64,74,75,82,84,87,88] (Table 1).

As previously mentioned, Wang and colleagues characterized plasma tsRNA signatures associated with breast cancer, developing a multivariate diagnostic model based on integrated plasma levels of tRF-Arg-CCT-017, tRF-Gly-CCC-001, and tiRNA-Phe-GAA-002. Moreover, elevated plasma levels of either tRF-Arg-CCT-017 or tiRNA-Phe-GAA-002 were indicative of shorter overall survival (OS) and disease-free survival (DFS) in patients with breast cancer [64]. Additionally, Jin et al. obtained the serum tsRNA expression profile related to pancreatic cancer and constructed a combined tsRNA diagnostic model incorporating the serum levels of tRF-Pro-AGG-004 and tRF-Leu-CAG-002. Using in situ hybridization, the authors also detected the expression levels of tRF-Pro-AGG-004 and tRF-Leu-CAG-002 in tumor tissues and paired normal tissues from patients with pancreatic cancer. The results indicated a significant upregulation of both tsRNAs in tumor tissues. Furthermore, higher expression levels of these tsRNAs were strongly correlated with reduced survival, and patients with concurrently elevated expression of both tsRNAs exhibited the shortest OS [60]. Li and colleagues profiled the salivary exosomal tsRNA landscape in ESCC and developed a combinatorial diagnostic model based on the salivary exosomal levels of tRNA-GlyGCC-5 and sRESE. Risk Score of Prognosis (RSP) was derived from the expression of these two markers, with high RSP correlating with diminished OS and progression-free survival (PFS). Further analysis underscored the therapeutic implications of RSP stratification, as high-RSP patients undergoing adjuvant therapy exhibited significantly prolonged OS and PFS. Collectively, these findings support the utility of the combined model in prognostic stratification and preoperative selection of patients likely to benefit from postoperative adjuvant treatment [74]. Yang et al. acquired the plasma tsRNA expression profile associated with NSCLC and identified AS-tDR-007333 as a candidate diagnostic biomarker. The authors leveraged fluorescence in situ hybridization to examine the expression of AS-tDR-007333 in tumor tissues, revealing a significant positive correlation between its high expression and reduced OS [61]. Ying et al. adopted an optimized RNA sequencing approach to analyze the tsRNA expression profiles of tumor tissues and matched adjacent normal tissues from three CRC patients. They identified 16 differentially expressed tsRNAs through comparative transcriptomic analysis. Subsequent qRT-PCR validation demonstrated significant downregulation of tRF3008A in tumor tissues. Notably, the elevated level of tRF3008A indicated longer DFS in CRC patients [82]. Utilizing an optimized RNA sequencing platform, Xiong and colleagues conducted comparative transcriptomic profiling of tsRNAs across matched sets of primary tumor tissues and synchronous hepatic metastases obtained from three patients with pancreatic cancer. This approach identified 226 differentially expressed tsRNAs. qRT-PCR validation further corroborated pronounced upregulation of tiRNA-Val-CAC-2 in hepatic metastases. Moreover, the authors detected the expression of tiRNA-Val-CAC-2 in serum samples from 40 patients with pancreatic cancer without distant metastasis and 30 patients with distant metastasis. tiRNA-Val-CAC-2 exhibited remarkably elevated level in the serum of patients with disseminated disease. Critically, elevated circulating levels of tiRNA-Val-CAC-2 correlated strongly with diminished OS [17].

## 4. tsRNAs as Potential Therapeutic Targets for Cancer

Researchers have discovered that a 3′-tsRNA derived from tRNA^Leu^, namely 3′-tsRNA^Leu^, enhanced the viability of HCC cells. Targeted silencing of 3′-tsRNA^Leu^ with antisense locked nucleic acid (LNA) oligonucleotides suppressed tumor growth in patient-derived xenograft (PDX) models of HCC [26,89]. Additionally, 5′-tRFCys was found to promote metastatic dissemination in breast cancer cells, and its targeted silencing via antisense LNA oligonucleotides reduced lung metastasis in patient-derived xenograft organoid (PDXO) models of breast cancer. These findings indicate that tsRNAs may serve not only as potential diagnostic and prognostic biomarkers for cancer but also as promising therapeutic targets for cancer treatment [20].

Multiple tsRNAs have been experimentally demonstrated to function as oncogenic promoters in nude mouse xenograft models. Functional inhibition of these tsRNAs may offer a novel therapeutic strategy for precision oncology [20,22,32,39,40,41,61,63,81,90] (Table 1). For instance, Yang et al. demonstrated that AS-tDR-007333 facilitated MED29 transcription through epigenetic modulation of its promoter region and potentiation of the transcription factor ELK4, thereby promoting proliferation and migration in NSCLC cells. Treatment with an AS-tDR-007333 inhibitor significantly reduced tumor volume and weight in nude mice models [61]. Furthermore, Tao and colleagues elucidated that hypoxia tumor induced the generation of 5′tiRNA-His-GTG in CRC cells via the HIF1α/angiogenin signaling axis. 5′tiRNA-His-GTG subsequently inhibited the Hippo signaling pathway through targeted inhibition of LATS2, consequently enhancing tumor proliferation and conferring resistance to apoptosis [39]. Consistent with these findings, administration of 5′tiRNA-His-GTG agomir increased tumor burden in vivo, whereas its antagomir attenuated tumor growth [39]. Similarly, Lu et al. reported that tRF-3022b enhanced the tumorigenicity of CRC by interacting with LGALS1 and MIF. Intervention with antisense LNA oligonucleotides of tRF-3022b led to marked suppression of tumor development in xenograft models [32]. In another study, Han et al. revealed that tiRNA-Gly promoted proliferation and migration in thyroid cancer through binding to RBM17 and modulating its dependent alternative splicing events. Silencing tiRNA-Gly with siRNA resulted in a remarkable reduction in tumor volume and weight in nude mice models [40]. Moreover, Cui and colleagues identified that tRF-Val directly bound to the chaperone protein EEF1A2, facilitating its nuclear translocation and interaction with MDM2. It further promoted malignant phenotypes in gastric cancer, including enhanced proliferation and invasion, and attenuated apoptosis in gastric cancer cells. Knockdown of tRF-Val using shRNA markedly diminished tumor weight and volume [22]. Additionally, Zhao et al. confirmed that tRF^Tyr^ activated lactate dehydrogenase A through direct binding, resulting in lactate accumulation and subsequently promotion of proliferation and metastasis in laryngeal squamous cell carcinoma. In vivo silencing of tRF^Tyr^ with shRNA effectively mitigated tumor volume [41]. Complementing these findings, Xiong et al. discovered that tiRNA-Val-CAC-2 bound to and stabilized FUBP1, prompting its enrichment at the C-MYC promoter and activating C-MYC transcription, which drove migration and invasion in pancreatic cancer. Treatment with tiRNA-Val-CAC-2 antagomir considerably reduced lung metastatic nodules in nude mice [17].

Beyond that, tsRNAs exhibiting tumor-suppressive functions may also serve as promising candidates for therapeutic intervention [29,42,43,45,82,90] (Table 1). Goodarzi and colleagues identified a subset of hypoxia-induced tsRNAs in breast cancer cells that competitively bound YBX-1, preventing its binding to the 3′UTRs of oncogenic transcripts. This interaction reduced mRNA stability and ultimately attenuated malignant phenotypes, including proliferation, migration, and invasion. In vivo, LNA-mediated suppression of these tsRNAs augmented pulmonary metastasis in nude mice, whereas synthetic tsRNA mimics significantly alleviated metastasis burden [29]. Additionally, Han et al. demonstrated that tRF3008A suppressed proliferation, migration, and invasion of colorectal cancer cells through downregulating FOXK1 expression and then inhibiting the Wnt/β-catenin signaling pathway. Consistent with its tumor-suppressive role, Inhibition of tRF3008A with antisense LNA oligonucleotides markedly accelerated tumor growth in xenograft models, while its overexpression with tRF3008A mimics significantly reduced tumor volume and lung metastasis [82]. Further supporting the therapeutic relevance of tumor-suppressive tsRNAs, Xu and colleagues showed that tRF-Val-CAC-016 acted through downregulation of CACNA1d and subsequent modulation of MAPK signaling to inhibit gastric cancer cell proliferation. Administration of tRF-Val-CAC-016 agomir resulted in significant reduction in tumor weight and volume in xenograft models [42].

Building upon the therapeutic potential of tsRNAs in directly modulating tumor growth and metastasis, it is noteworthy that emerging evidence also implicates specific tsRNAs in mediating resistance to conventional cancer therapies, including chemotherapy and radiotherapy [91,92,93,94]. For instance, Mo et al. employed high-throughput sequencing to identify the tsRNA expression profile in adriamycin-resistant breast cancer cells and further demonstrated that 3′tRF-Ala-AGC confers chemoresistance in breast cancer cells by modulating M2 macrophage polarization via TRADD signaling [91]. Targeting these therapy-resistant tsRNAs could thus not only inhibit tumor progression directly but also sensitize cancer cells to established treatment modalities.

## 5. Conclusions and Prospects

As an emerging class of sncRNAs, tsRNAs exhibit considerable promise in clinical diagnosis, prognosis assessment, and targeted therapy for cancer. Nevertheless, their translation into clinical applications is confronted with substantial technical challenges. The high degree of modification endows tsRNAs with exceptional stability in biofluids, making them promising biomarkers for liquid biopsy. Paradoxically, these same modifications hinder the comprehensive capture of tsRNA expression profiles using conventional sequencing technologies, thus currently limiting their utility as ideal biomarkers. Although several research groups have attempted to optimize sequencing strategies through enzymatic removal of methyl modifications to obtain more complete tsRNA expression profiles [35,36,37,38,95,96,97], these efforts remain inadequate for resolving the diverse repertoire of rare and modified nucleotides inherent to tsRNAs. Moreover, since tsRNA modifications are closely related to their higher-order structures, stability, and functions, it is the naïve, modified tsRNAs, rather than “naked” ones, that must be characterized. Currently, high-throughput technologies capable of large-scale mapping of tsRNA modifications are under development, including MS-based direct sequencing and nanopore-based direct sequencing [9]. Deciphering the “code” of the tsRNA modifications is imperative to unravel their biological functions and evaluate their value as therapeutic targets.

In spite of these challenges, the field has matured significantly, supported by the development of dedicated computational resources. A suite of online databases and tools, such as tRF2Cancer, MINTbase v.2.0, tRFexplorer, concotRF, tsRBase, Pol3Base, tsRFun, and tsRNADisease, has been established to facilitate tsRNA identification, quantification and functional exploration in cancer [98,99,100,101,102,103,104]. These resources collectively empower researchers to mine tsRNA candidates in further cancer research.

## Data Availability

This review is based on previously published studies and publicly available resources. No new data or materials were generated or analyzed in this study.

## Abbreviations

The following abbreviations are used in this manuscript:

AML	Acute myeloid leukemia
AUC	Area under the curve
CA19-9	Carbohydrate Antigen 19-9
CACNA1d	Calcium voltage-gated channel subunit alpha1 D
CEA	Carcinoembryonic Antigen
CLL	Chronic lymphocytic leukemia
C-MYC	MYC proto-oncogene, bHLH transcription factor
CRC	Colorectal cancer
CT	Computed Tomography
DFS	Disease-free survival
EEF1A2	Eukaryotic translation elongation factor 1 alpha 2
ESCC	Esophageal squamous cell carcinoma
EVs	Extracellular vesicles
FOXK1	Forkhead box K1
FUBP1	Far upstream element binding protein 1
HCC	Hepatocellular carcinoma
LATS2	Large Tumor Suppressor Kinase 2
LGALS1	Galectin 1
LNA	Locked nucleic acid
LSCC	Laryngeal squamous cell carcinoma
MDM2	MDM2 proto-oncogene
miRNA	microRNA
MM	Multiple myeloma
mRNA	Messenger RNA
NSCLC	Non-small cell lung cancer
OS	Overall survival
PBMCs	Peripheral blood mononuclear cells
PDX	Patient-derived xenograft
PDXO	Patient-derived xenograft organoid
PFS	Progression-free survival
pre-tRNAs	Precursors tRNAs
qRT-PCR	quantitative reverse-transcription PCR
RBM17	RNA binding motif protein 17
RCC	Renal cell carcinoma
RG4	RNA G-quadruplex
RSP	Prognostic risk score
sncRNAs	Small non-coding RNAs
TCL1	T-Cell leukemia/lymphoma 1
tRNAs	Transfer RNAs
tsRNAs	Transfer RNA-derived small RNAs
UTR	Untranslated region
YBX-1	Y-box binding protein 1
